# Identification of ^18^F-FDG PET/CT Parameters Associated with Weight Loss in Patients with Esophageal Cancer

**DOI:** 10.3390/nu15133042

**Published:** 2023-07-05

**Authors:** Thierry Galvez, Ikrame Berkane, Simon Thézenas, Marie-Claude Eberlé, Nicolas Flori, Sophie Guillemard, Alina Diana Ilonca, Lore Santoro, Pierre-Olivier Kotzki, Pierre Senesse, Emmanuel Deshayes

**Affiliations:** 1 Department of Nuclear Medicine, Institut du Cancer de Montpellier, Université de Montpellier, 34298 Montpellier, Cedex 5, France; 2 Department of Endocrinology, Diabetes and Nutrition, CHU de Montpellier, Université de Montpellier, 34295 Montpellier, France; 3 Biometry Unit, Institut du Cancer de Montpellier, Université de Montpellier, 34298 Montpellier, Cedex 5, France; 4 Department of Clinical Nutrition and Gastroenterology, Institut du Cancer de Montpellier, Université de Montpellier, 34298 Montpellier, Cedex 5, France; 5 Institut de Recherche en Cancérologie de Montpellier (IRCM), INSERM U1194, Université de Montpellier, 34298 Montpellier, France

**Keywords:** malnutrition, ^18^F-FDG PET/CT, weight loss, brain metabolism, esophageal cancer

## Abstract

^18^F-FDG PET-CT is routinely performed as part of the initial staging of numerous cancers. Other than having descriptive, predictive and prognostic values for tumors, ^18^F-FDG PET-CT provides full-body data, which could inform on concurrent pathophysiological processes such as malnutrition. To test this hypothesis, we measured the ^18^F-FDG uptake in several organs and evaluated their association with weight loss in patients at diagnosis of esophageal cancer. Forty-eight patients were included in this retrospective monocentric study. ^18^F-FDG uptake quantification was performed in the brain, the liver, the spleen, bone marrow, muscle and the esophageal tumor itself and was compared between patients with different amounts of weight loss. We found that Total Lesion Glycolysis (TLG) and peak Standardized Uptake Values (SUV_peak_) measured in the brain correlated with the amount of weight loss: TLG was, on average, higher in patients who had lost more than 5% of their usual weight, whereas brain SUV_peak_ were, on average, lower in patients who had lost more than 10% of their weight. Higher TLG and lower brain SUV_peak_ were associated with worse OS in the univariate analysis. This study reports a new and significant association between ^18^F-FDG uptake in the brain and initial weight loss in patients with esophageal cancer.

## 1. Introduction

In oncology, 2-deoxy-2-(^18^F)-fluoro-D-glucose positron emission tomography coupled to computed tomography (^18^F-FDG PET-CT) is widely used in order to detect regional and distant tumor spread as part of the initial tumor staging or treatment evaluation [1]. Specific metrics based on ^18^F-FDG uptake at the tumor level are also recognized as independent prognostic factors in several cancers, including esophageal cancer [2,3,4]. However, ^18^F-FDG uptake by neoplastic or non-neoplastic tissues, though measurable, has only rarely been used to explore concurrent pathophysiological processes, such as malnutrition [5,6]. Cancer-associated malnutrition is a severe systemic metabolic condition, with a high incidence in patients with esophageal cancers [7,8]. It is defined by involuntary WL, low body mass index (BMI) or reduced muscle mass in the context of reduced food intake, reduced nutrient absorption or active disease [9]. It impairs quality of life, associates with worse tolerance to treatment and negatively impacts overall survival (OS) [10,11,12]. How malnutrition impacts ^18^F-FDG uptake of tumors and healthy organs is largely unknown [13,14]. Likewise, to what extent ^18^F-FDG uptake could inform the pathophysiological changes occurring in malnourished patients is also unknown.

In this study, using data from routinely performed ^18^F-FDG PET-CT at the initial staging of esophageal cancer, we retrospectively and systematically assessed the association of ^18^F-FDG uptake values in the brain, the liver, the spleen, bone marrow, muscle and the esophageal tumor itself with weight loss. 

## 2. Methods

### 2.1. Patient Selection

Patients aged 18 years old or above diagnosed with esophageal cancer (squamous cell carcinoma or adenocarcinoma) that underwent an ^18^F-FDG PET-CT scan for initial staging (before any treatment) between January 2014 and June 2019 were eligible for inclusion. The study was approved by the Institutional Review Board (ART-2021-02). Patients were excluded if anti-diabetic medications were listed in their medical files, if their capillary blood glucose concentration at the time of the ^18^F-FDG injection was below 65 mg/mL or above 135 mg/mL, if the time between tracer injection and imaging was under 55 min or above 75 min, if their brain had not been scanned, if they presented with brain, liver or spleen metastases or if CT and PET images were misaligned at visual inspection. 

### 2.2. Imaging Data Acquisition and Processing

Patients were asked not to ingest anything other than plain water and to avoid intense physical activity for 6 h before the injection of ^18^F-FDG (3.5 MBq/kg). Their venous blood glucose level was measured before injection using a glucometer. Image acquisition from skull to mid-thigh was performed on the same Discovery PET/CT 690 scanner (GE Healthcare, Waukesha, WI, USA). Non-contrast CT scans of patients in the supine position were acquired, followed by 3D PET imaging. Data were corrected for geometrical response and detector efficiency, dead time, random coincidences, scatter and attenuation, as recommended in [15], and reconstructed into matrices of 256 × 256 pixels. Our PET/CT imaging facility was accredited for tumor imaging by the European Association of Nuclear Medicine Research Ltd.

### 2.3. Quantification of ^18^F-FDG Uptake

The quantification of ^18^F-FDG uptake was retrospectively performed. Spherical volumes of interest (VOI) were manually positioned over relevant organs using CT images and OsiriX MD software (version 7.5): over the right lobe of the liver (19.2 cm^3^), over the spleen (5.2 cm^3^), inside the brain (centered on the putamen) and inside the left iliac tuberosity in order to measure tracer uptake in the bone marrow. The putamen is an easily recognizable brain structure, which we used to reproducibly center the brain VOI. This warranted consistent measurements. SUV_peak_ were computed within these VOI using OsiriX MD. SUV_peak_ correspond to the average value within a 1 cm^3^ sphere positioned around the highest voxel value (SUV_max_) [15,16,17]. SUV_peak_ were proposed to be more robust than SUV_max_, especially in low-count conditions, as was the case for most organs in this study [18]. The esophageal tumor was circumscribed within a large spherical VOI. The Metabolic Tumor Volume (MTV) was defined as the volume inside the 3D isocontour at 41% of the maximum pixel value (as recommended in [15]) and the Total Lesion Glycolysis (TLG) as MTV multiplied by mean voxel SUV (SUV_mean_) within the MTV. For skeletal muscle, the mean SUV (SUV_mean_) of a 2D, manually drawn region of interest (ROI) delineating the cross-sectional area of skeletal muscle at the third lumbar vertebra was chosen. This region has been shown to be representative of whole-body muscle mass [19]. When indicated, SUVs were normalized to lean body mass (LBM) according to James’ and Janmahasatian’s predictive equations [20,21,22]) and referred to as SUL_James_ or SUL_Janma_, respectively. Similarly, when indicated, brain SUV_peak_ were also normalized to blood glucose concentrations at the time of the ^18^F-FDG injection as SUV_glu_ = SUV_peak_ × (blood glucose in mg/dL)/100) [23]. 

### 2.4. Clinical Data Collection and Nutritional Assessment

Clinical parameters and imaging conditions were obtained from patients’ medical records, PET/CT reports and associated DICOM files. The reference weight (weight[ref]) was defined as the patient-reported usual stable weight. Weight loss (WL) was defined as: WL = ($weight\left[PET\right]-weight\left[ref\right])/weight\left[ref\right]$ with weight[PET] defined as the weight on the day of PET/CT. WL was categorized according to two thresholds: WL ≥ 5% and WL ≥ 10%. The reference weight was obtained from nutritional reports systematically filed for all patients by dieticians or physicians during consultation. 

### 2.5. Statistical Analysis

Categorical variables are expressed as numbers in the indicated category and (%), with continuous variables as median and (range). Group differences between quantitative variables were tested using the non-parametric Kruskal-Wallis test by ranks or Pearson’s chi-square test for categorical variables. In order to examine the optimal cut-off values for SUVs, Receiver Operating Characteristic (ROC) curves were assessed with WL ≥ 10% as the reference. The cut-off value corresponding to the highest predictive value, which maximized the Youden index, was chosen. Overall survival (OS) was defined as the time between diagnosis and death or last follow-up (censored data). OS was estimated using the Kaplan-Meier estimator. The log-rank test was performed to assess differences between groups. Patients alive without event were censored at the last news date. The median follow-up was estimated according to «reverse Kaplan-Meier method» and presented with 95% confidence intervals (CIs). Multivariate analyses were carried out using logistic regressions or Cox’s proportional hazards regressions, with a stepwise selection procedure on covariables with *p* < 0.1 (dichotomized at median value) in univariate analyses. We added 3 more variables of interest, i.e., Glycemia, TLG and MTV, which were not automatically selected as categorical variables for the multivariate logistic regression, but were associated with WL ≥ 10% (*p* < 0.1) as continuous variables. Odds ratio (OR) and hazard ratios (HR) are presented with 95% CIs. All *p* values reported were two-sided and the significance level was set to 5% (*p* < 0.05) and indicated by *. Statistical analysis was performed using the STATA 16.1 software (Stata Corporation, College Station, TX, USA).

## 3. Results

### 3.1. Demographic and Nutritional Characteristics of Patients

Two hundred and eighteen patients with esophageal cancer underwent an initial ^18^F-FDG PET-CT scan in our institute between January 2014 and June 2019. One hundred and fifty-three patients were excluded because the PET-CT scans did not encompass their brain or showed improper alignment between the PET and CT images in the brain area; thirteen patients were excluded because of known diabetes or blood glucose outside of the 65–135 mg/L range; four patients were excluded because the time between the ^18^F-FDG injection and imaging was outside the predefined range. Forty-eight patients were selected for the study. The median usual BMI was 27.2 kg·m^−2^ before the onset of initial symptoms. In comparison to the usual weight, the median WL was 7% on the PET scan day. Thirty-two persons (67%) lost 5% or more of their usual weight and eighteen (37.5%) lost 10% or more of their usual weight. Values of BMI and glycemia before PET imaging were significantly different between patients who lost 10% or more of their initial weight compared to the rest of the cohort (Table 1).

### 3.2. TLG and Brain SUV_peak_ Associated with WL ≥ 10%

**Figure 1:**
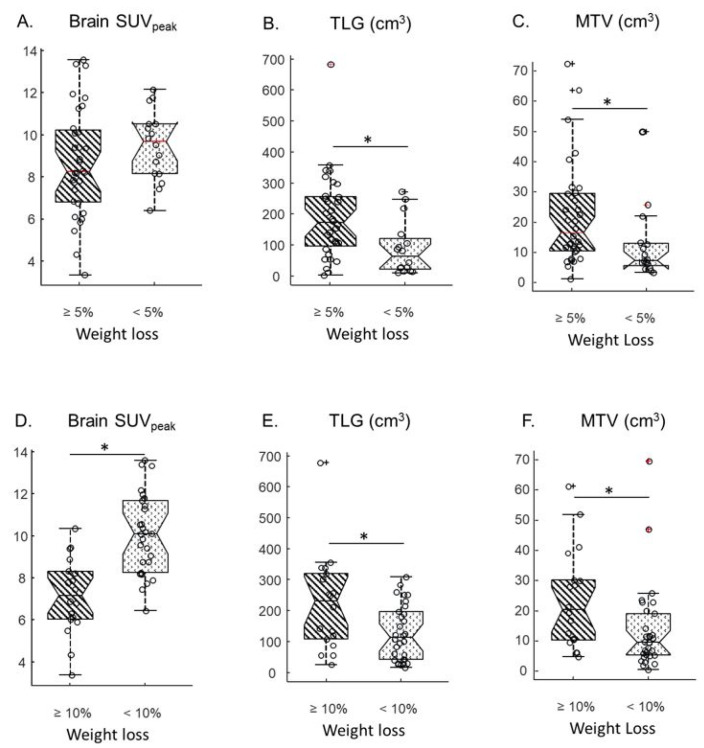
Comparison of brain SUV_peak_ (**A**), Total Lesion Glycolysis (TLG) (**B**) and Metabolic Tumour Volume (MTV) (**C**) from patients who lost 5% or more of their initial weight (diagonal lines) versus patients who lost less than 10% of their initial weight (dots). Comparison of brain SUV_peak_ (**D**), TLG (**E**) and MTV (**F**) from patients who lost 10% or more of their initial weight (diagonal lines) versus patients who lost less than 10% of their initial weight (dots). Circles represent data points. Central horizontal marks correspond to medians; bottom and top edges of the box indicate the 25th and 75th percentiles, respectively; notches correspond to limits of 95% CI. Whiskers extend to the most extreme data value that is not beyond +/−2.7σ. Crosses correspond to data points beyond whiskers. * indicates *p* < 0.05.

**Figure 2:**
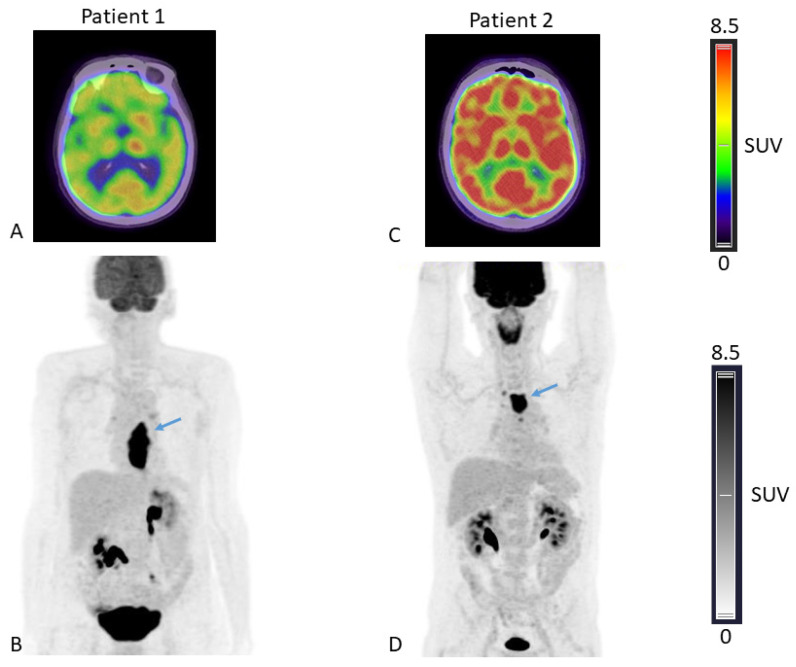
^18^F-FDG-fused PET/CT axial slices passing through the brain (**A**,**C**) and Maximal Intensity Projection, anterior view (**B**,**D**), of patient 1 presenting with low brain ^18^F-FDG uptake (brain SUV_peak_ = 5.89) and a body weight loss of 10% compared to usual weight and patient 2 presenting with higher brain ^18^F-FDG uptake (brain SUV_peak_= 10.17) and a body weight loss of 8% compared to usual weight. Arrows depicting oesophageal tumors in both patients.

The SUV_peak_ measured in the brain, the liver, the spleen, bone marrow, muscle and primary tumor were compared between patients presenting with WL ≥ 5% versus <5% on one hand, and WL ≥ 10% versus <10% on the other hand. When a cut-off of 5% WL was chosen, no significant difference was observed between SUV_peak_ from any organs (Table 2, columns 3–5 and Figure 1A). Yet, when a cut-off value of 10% WL was used, the brain SUV_peak_ were significantly lower (*p* < 0.001, Kruskal-Wallis test) in patients who lost 10% or more of their usual weight compared to other patients (Table 2, columns 6–8 and Figure 1D). The Spearman correlation coefficient between the brain SUV_peak_ and weight difference was −0.44, *p* = 0.0015 (Supplementary Figure S1A). Representative ^18^F-FDG PET-CT images from two patients presenting with different levels of brain ^18^F-FDG uptake are shown (Figure 2).

No significant difference was observed in SUV_peak_ for the spleen, bone marrow muscle or primary tumor using a WL cut-off of 10% (Table 2). For primary tumors, the median MTV and TLG were both significantly higher in patients that met either the 5% WL cut-off or the 10% WL (Figure 1B,C,E,F). The Spearman correlation coefficient between TLG and weight difference was −0.48, *p* < 0.001 (Supplementary Figure S1B). There was no significant correlation between TLG and brain SUV_peak_ (Spearman correlation coefficient of −0.23, *p* > 0.1, Supplementary Figure S1C).

Patient BMI and glycemia were potential confounding factors as both are known to influence SUVs [21,23,24,25,26,27] and distributions of both parameters were significantly different between patients who lost 10% or more of their usual weight and the others (Table 1). However, in a multivariate logistic regression model to predict WL ≥ 10%, adjusted for BMI, glycemia, brain SUV_peak_, MTV and TLG, only brain SUV_peak_ and TLG remained significant predictors of WL ≥ 10% (Table 3). Moreover, using brain SUV_peak_ normalized by lean body weight (i.e., SUL) or by glycemia (i.e., SUV_glu_) did not change the results: brain SUL or brain SUV_glu_ were lower in patients who lost 10% or more of their usual weight (Supplementary Table S1).

A cut-off value of 7.32 for brain SUV_peak_ determined with the analysis of the ROC curve (AUC 0.863) was able to predict WL ≥ 10% with a high specificity of 0.97, but with a low sensitivity 0.53. Similar trends were observed with ROC analysis of TLG but AUC was lower; i.e., 0.743 (Table 4).

### 3.3. TLG and Brain SUV_peak_ Associated with Survival

**Figure 3:**
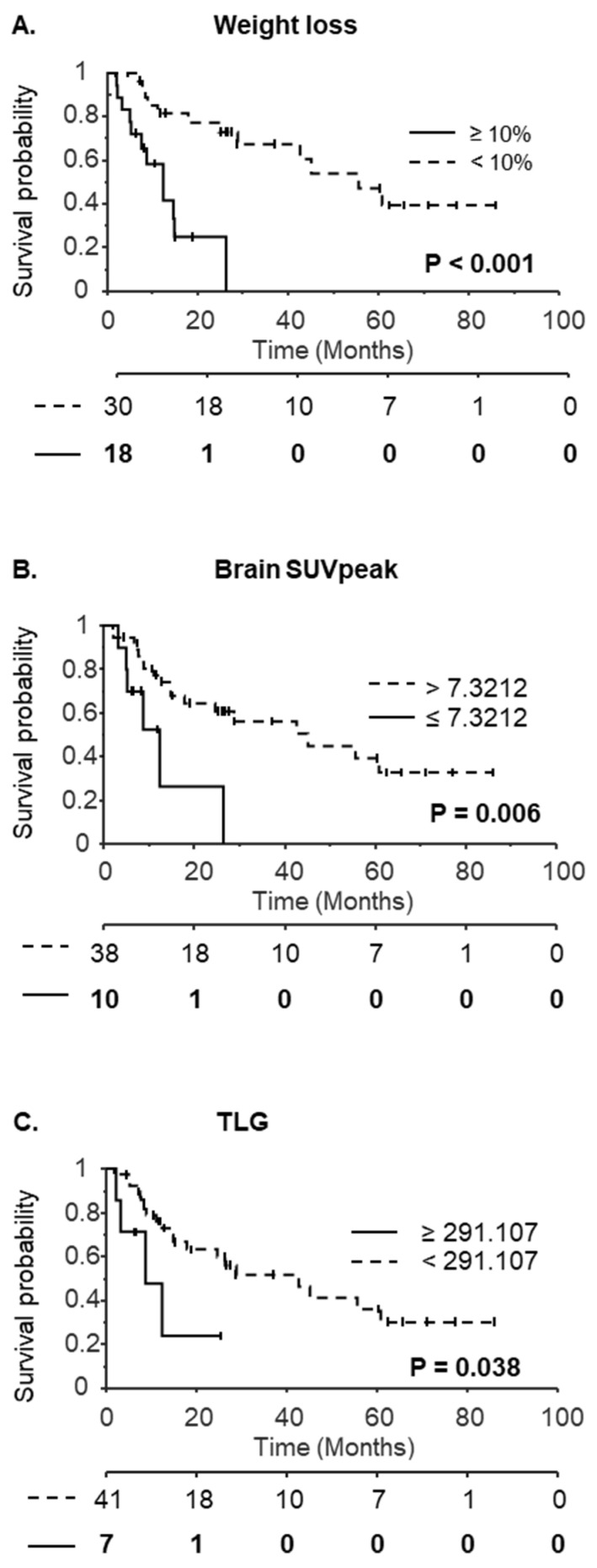
Kaplan-Meier curves with respect to weight loss ≥ 10% (**A**), brain SUV_peak_ (**B**) or Total Lesion Glycolysis (TLG) (**C**). The number at risk is indicated below the x-axis. For brain SUV_peak_ and TLG, groups of patients were defined according to cut-off values determined by the ROC analysis.

The median follow-up period was 28.7 months. Using Kaplan Meier analysis and groups split at the median value, none of the PET variables significantly affected OS. Only the presence of distant metastasis, BMI on the day of the PET scan and WL ≥ 10% were prognostic factors (Table 5, Figure 3A). When cut-off values determined by ROC analysis to predict WL were used (Table 5), both brain SUV_peak_ and TLG were significant prognostic factors (Table 5, Figure 3B,C).

In a cox multivariate model (Log likelihood = −67.31) including all variables with *p* < 0.1 in cox univariate analysis (i.e., distant metastasis, BMI on the day of the PET scan, WL ≥ 10%, brain SUV_peak_, MTV and TLG), only the presence of distant metastasis and WL ≥ 10% were associated with overall survival (HR = 2.97, *p* = 0.046 and HR = 4.35, *p* = 0.015 respectively), indicating that brain SUV_peak,_ and TLG are not independent prognostic factors. 

## 4. Discussion

Using data obtained from routine ^18^F-FDG PET-CT, we showed that, in patients at diagnosis of esophageal cancer, WL correlated with high TLG but also with low brain SUV_peak_. In addition, in the univariate analysis, both TLG and brain SUV_peak_ were pre-therapeutic prognostic factors in these patients, possibly in connection with weight loss. In this group of patients, weight loss did not associate with SUV_peak_ measured in the liver, the spleen, bone marrow or muscle. This specificity of the brain results cannot be simply explained by the higher amplitude of the signal observed in the brain, as tumor SUVs are similarly high and do not differ between WL categories. 

WL ≥ 5% within the past 6 months or WL ≥ 10% beyond 6 months defines malnutrition in the context of cancer [9]. Lower brain SUVs are specifically associated with more pronounced WL, i.e., ≥10%, whereas higher TLG is associated with both high and more moderate WL; i.e., ≥5%. A recent study has shown a significant association between esophageal tumor SUV_max_ and weight loss. Although we found a similar association between TLG and weight loss, SUV_peak_ were not significantly associated with weight loss in our group of patients [13]. To our knowledge, this is the first clinical report of the association of a routine ^18^F-FDG uptake measurement in the brain with malnutrition and survival in patients just diagnosed with esophageal cancer. It was made possible because of the unique and systematic survey and filing of patients’ weight history by dedicated dieticians in our clinical center [24]. In a cachexia-inducing murine model of adenocarcinoma, brain uptake was significantly higher in cachexic mice compared to the group of non-cachexic mice; the reason for this discrepancy with our results is unclear, but it may be explained by the inherent limitations of the preclinical model when compared to the patients [25].

The association of weight loss with high TLG may be explained by the higher metabolic burden of a high-volume tumor, as well as by the larger hindrance of such a tumor on the esophagus, hence limiting food intake. It is more difficult to explain the correlation between weight loss and brain SUV_peak_. The SUV of a given organ is key as the brain depends on its intrinsic metabolic properties, but also on several other parameters [26]. Systemic changes in body composition, especially changes in the fraction of fat mass, may indeed affect the biodistribution of the tracer. For instance, liver, blood and spleen SUVs are overestimated in obese persons compared to non-obese persons [22,27,28]. The normalization of SUVs by lean mass was introduced in order to circumvent this effect. The opposite phenomenon, i.e., the underestimation of tissue SUVs, may explain our results in undernourished patients who might have lost more fat than lean mass. However, lower SUVs were specifically observed in the brains of undernourished persons and not in other tissues. After normalization to lean mass estimated by predictive equations [20,21], brain SUVs were still lower in patients who lost ≥ 10% of their initial weight (Supplementary Table S1). One cannot exclude that ^18^F-FDG uptake in voluminous tumors may reduce the amount of ^18^F-FDG available for uptake in the brain, but, in this case, a similar trend should have been observed in other tissues [29]. Moreover, we did not find any correlation between TLG and brain SUV_peak_ (Supplementary Figure S1C).

The blood glucose concentration may also affect SUVs, as endogenous glucose competes with the tracer and brain SUVs are known to be highly sensitive to glycemia [30,31,32]. In our group of patients, the blood glucose concentration was slightly higher in patients who lost 10% or more of their weight compared to patients who lost less than 10% of their weight (Table 1), indicating glycemia to be a possible confounding factor. However, brain SUVs reduction persisted in multivariate analysis after adjustment for glycemia, sex or age. In addition, when corrected for blood glucose [22], brain SUV_glu_ were still significantly lower in patients who lost 10% or more of their weight (Supplementary Table S1). 

The pathophysiology underlying the reduced ^18^F-FDG uptake in the brain of undernourished patients is unknown. The brain relies almost exclusively on glucose as an energy source and reduced cerebral glucose metabolism may be an adaptive mechanism to reduced nutrient availability. In agreement with this hypothesis, starvation was shown to be associated with decreased glucose consumption, specifically in the brain [33,34,35], and glycolytic flux and phosphofructokinase activity were significantly reduced in the neurons of starved mice [36]. Instead of glucose, neurons have been proposed to use ketone bodies as complementary fuel, which may decrease brain glucose uptake [36,37,38,39,40].

A decrease in brain SUV_peak_ was only observed with WLs ≥ 10%, which corresponds to stage 2/severe malnutrition [9]. Severe malnutrition may indeed correspond to extreme metabolic states, e.g., starvation and ketogenesis (cf above), which are associated with brain hypometabolism. A lesser weight loss may not trigger such a metabolic switch. 

Several medical conditions, especially in neurology and psychiatry, have been shown to be associated with changes in ^18^F-FDG uptake in the brain. Alzheimer’s disease is associated with low ^18^F-FDG uptake in specific regions of the brain depending on the severity and the duration of the disease [41]. ^18^F-FDG uptake is also lower in the frontal cortex of schizophrenia patients [42] or in the thalami of patients with delirium [43]. It is unknown whether and how these observations relate to the lower ^18^F-FDG uptake described here, but the prevalence of mood disorders is high among patients with esophageal cancer, impacting their quality of life and pain perception [44,45].

As shown by others and confirmed in this study, WL is a strong prognostic factor in esophageal cancer. In our population, brain SUV_peak_ and TLG were also pre-therapeutic prognostic factors when cut-off values predictive of WL were used in the univariate analysis. TLG has already been identified as a prognostic factor, but not brain SUV_peak_ [46]. Their prognostic value is lost in multivariate Cox models, suggesting that brain SUV_peak_ or TLG are not independent prognostic factors and affect survival because of their association with other factors; e.g., WL. 

This study has several limitations: it was retrospective, performed at a single clinical center and, above all, included a small number of patients, mostly as a consequence of the exclusion criteria requiring the inclusion of the brain in the full body scan. Additional work on a larger cohort will be necessary to confirm our results. Moreover, though statistically significant, data supporting the prognostic value of brain SUV_peak_ and TLG relied on a small number of patients, especially within the group with the lowest OS (Figure 2), and must be confirmed with a larger group of patients. The clinical significance of our findings is not yet clear. Although brain SUV_peak_ are indicative of severe weight loss, they are obviously not a substitute for the clinical approach; i.e., taking the patient’s actual weight and history. However, low brain SUV_peak_ could trigger nutritional assessment if it has not been carried out at the time of the PET scan. 

This work revealed a so far unnoticed association between malnutrition and routine ^18^F-FDG uptake measurements in the tumors and, more surprisingly, brains of patients diagnosed with esophageal cancer. It may open up new avenues of research aimed at understanding the systemic consequences of malnutrition, especially on the central nervous system and its cognitive and behavioral functions.

## Figures and Tables

**Table 1:** Distribution of patient characteristics according to weight loss. Fractional weight difference is between the usual weight, stable weight and the weight measured just before PET imaging. Median and (range) are indicated. *p* is according to Kruskal-Wallis test by rank for continuous variables and to Pearson’s chi-square test for categorical variables.

Variable		All	% WL ≥ 5	% WL < 5	*p*	% WL ≥ 10	% WL < 10	*p*
Number of patients (%)		48	32 (67)	16 (33)		18 (37.5)	30 (62.5)	
Number of females (%)		8 (16.7)	5 (16)	3 (19)		3 (17)	5 (17)	
Age at diagnosis, years		64 (36:88)	61.5 (48.0:82.0)	67.0 (36.0:88.0)	0.251	61.0 (54.0:82.0)	66.5 (36.0:88.0)	0.273
Usual BMI, kg·m^−2^		27.2 (17.0:40.8)	27.6 (17.0:40.8)	25.7 (20.0:40.3)	0.718	27.2 (17.0:33.3)	27.0 (20.0:40.8)	0.647
BMI on PET scan day, kg·m^−2^		24.6 (14.1:38.5)	24.3 (14.1:36.9)	25.4 (19.4:38.5)	0.088	22.1 (14.1:28.7)	25.6 (19.4:38.5)	0.004 *
Fractional weight difference		−0.07 (−0.42:08)	−0.11 (−0.4:−0.1)	0.00 (−0.0:0.1)		−0.14 (−0.4:−0.1)	−0.04 (−0.1:0.1)	<0.001 *
Number of Histological type (%)	Squamous cell carcinoma	28 (58)	17 (53)	11 (69)	0.300	11 (61)	17 (57)	0.762
	Adenocarcinoma	20 (42)	15 (47)	5 (31)		7 (39)	13 (43)	
Patients with distant metastasis		7	5	2	0.772	4	3	0.245
History of former cancer (%)		14 (29)	6 (19)	8 (50)	0.042 *	4 (22)	10 (33)	0.412
Time tracer injection–PET acquisition, min.		63.0 (55.0:73.0)	64.0 (55.0:73.0)	59.0 (55.0:68.0)	0.024 *	62.5 (55.0:71.0)	63.0 (55.0:73.0)	0.958
Glycemia before PET, mg/dL		99.5 (65:134)	101.0 (65.0:134.0)	97.5 (84.0:131.0)	0.550	103.0 (90.0:134.0)	97.5 (65.0:131.0)	0.023 *

* indicated *p* < 0.05.

**Table 2:** Distribution of ^18^F-FDG uptake values in specified organs according to weight loss (WL). MTV, Metabolic Tumor Volume; TLG, Tumor Lesion Glycolysis. Median and (range) are indicated. *p* is according to Kruskal-Wallis test by rank.

		All	% WL ≥ 5	% WL < 5	*p*	% WL ≥ 10	% WL < 10	*p*
Brain	SUV_peak_	8.8 (3.4:13.6)	8.3 (3.4:13.6)	9.7 (6.4:12.2)	0.189	7.2 (3.4:10.3)	10.1 (6.4:13.6)	<0.001 *
Liver	SUV_peak_	2.8 (1.4:4.5)	2.8 (1.4:4.5)	2.0 (1.6:2.4)	0.710	2.8 (1.4:3.4)	2.8 (2.2:4.5)	0.148
Spleen	SUV_peak_	2.3 (1.4: 3.9)	2.4 (1.9:2.9)	1.7 (1.2:2.1)	0.670	2.2 (1.4:2.7)	2.4 (1.8:3.9)	0.170
Bone marrow	SUV_peak_	1.7 (1.0:3.5)	1.7 (1.0:3.5)	1.6 (1.0:2.4)	0.678	1.7 (1.0:2.4)	1.7 (1.0:3.5)	0.307
Muscle at L3	SUV_mean_	0.7 (0.5:1.6)	0.7 (0.5:0.9)	0.7 (0.5:1.6)	0.623	0.7 (0.6:0.9)	0.7 (0.5:1.6)	0.221
	SUV_peak_	11.4 (1.8:28.7)	12.1 (1.8:28.7)	10.6 (3.7:22.4)	0.431	14.2 (3.9:28.7)	11.1 (1.8:22.4)	0.394
Primary tumor	MTV (cm3)	11.4 (0.5:69.6)	15.4 (0.5:69.6)	5.7 (1.9:47.0)	0.003 *	20.3 (4.6:61.1)	9.6 (0.5:69.6)	0.013 *
	TLG	125.8 (3.0:677.8)	171.3 (3.0:677.8)	63.7 (11.1:269.9)	0.005 *	230.1 (24.6:677.8)	99.9 (3.0:295.4)	0.005 *

* indicated *p* < 0.05.

**Table 3:** Multivariate logistic regression predicting weight loss ≥ 10%. Covariables with *p* < 0.1 in univariate analysis were chosen as adjustment variables, i.e., age at diagnosis (categorical), BMI on TEP scan day (categorical), Brain SUV_peak_ (categorical), Spleen SUV_peak_ (categorical), Liver SUV_peak_ (categorical), Glycemia before PET (mg/dL) (continuous), TLG (continuous), MTV (continuous).

		Odds Ratio	*p* > |z|	95% CI
TLG		1.004	0.031	1.000–1.009
Brain SUV_peak_				
	<8.82 (median)	1		
	≥8.82	0.098	<0.001	0.028–0.346

**Table 4:** ROC analysis of PET variables associated with weight loss. AUC, area under the ROC curve.

**Variables**	**AUC**	**Optimal Cut-Point Value**	**# Patients above Cut-Point**	**AUC**	**Optimal Cut-Point Value**	**# Patients above Cut-Point**
	WL ≥ 5%			WL ≥ 10%		
Brain SUV_peak_	NA			0.863	7.32	10
MTV	0.763	12.03	21	0.717	40.78	5
TLG	0.748	107.86	27	0.743	291.1	7

**Table 5:** Univariate Kaplan Meier analysis of overall survival. Cut-offs defining groups of patients are median unless otherwise specified; for variables predictive of 10%WL in multivariate logistic regression, cut-offs were determined by ROC analysis. Median are indicated in Table 1, column 2. HR: Hazard Ratio; CI: Confidence Interval.

Variables	Cut-Off	HR	95% CI	*p*
Glycemia before PET (mg/dL)	99.5 (median)	1.82	[0.801–4.12]	0.137
Sex (female)	yes/no	1.82	[0.546–6.07]	0.218
Age at diagnosis	64 (median)	0.902	[0.405–2.01]	0.798
Distant metastasis	yes/no	3.77	[0.871–16.3]	0.002 *
History of former cancer	yes/no	0.975	[0.406–2.34]	0.954
Usual BMI, kg·m^−2^	27.2 (median)	1.21	[0.543–2.71]	0.631
BMI (on the day of PETscan), kg·m^−2^	24.6 (median)	0.409	[0.181–0.924]	0.0268 *
Weight loss	≥5% vs. <5%	2.19	[0.98–4.94]	0.083
	≥10% vs. <10%	4.17	[1.52–11.5]	5.88 × 10^−5^ *
Brain SUV_peak_	8.8 (median)	0.634	[0.274–1.47]	0.241
	≤7.32 vs. >7.32	0.31	[0.0785–1.22]	0.0065 *
Liver SUV_peak_	2.8 (median)	1.08	[0.484–2.43]	0.844
Spleen SUV_peak_	2.3 (median)	1.16	[0.52–2.57]	0.722
Bone marrow SUV_peak_	1.7 (median)	1.11	[0.496–2.48]	0.798
Muscle SUV_mean_	0.7 (median)	1.29	[0.581–2.88]	0.528
Primary tumor SUV_peak_	11.4 (median)	0.954	[0.428–2.13]	0.908
MTV	11.4 (median)	2.15	[0.96–4.82]	0.0616
TLG	125.8 (median)	1.68	[0.736–3.85]	0.192
	≤291 vs. >291	2.89	[0.568–14.7]	0.038 *

* indicated *p* < 0.05.

## Data Availability

The datasets analyzed during the current study are available from the corresponding author on reasonable request.

## Supplementary Materials

The following supporting information can be downloaded at: https://www.mdpi.com/article/10.3390/nu15133042/s1, Figure S1: Correlation between brain SUVpeak and fractional weight difference (A). Correlation between Total Lesion Glycolysis (TLG) and fractional weight difference (B). Correlations beween brain SUVpeak and TLG (C). Spearman correlation coefficient and *p*-value of observing the null hypothesis. Table S1: Brain ^18^FDG uptake values normalized to lean body mass or blood glucose according to weight loss.
