# Supplementary material for: Identification of 18F-FDG PET/CT Parameters Associated with Weight Loss in Patients with Esophageal Cancer
Source: Nutrients. 2023 Jul 5;15(13):3042. doi: 10.3390/nu15133042 (PMC10346913; doi:10.3390/nu15133042)
Supplement: Supplementary file 1 [file nutrients-15-03042-s001.zip › nutrients-2414325-supplementary.pdf]

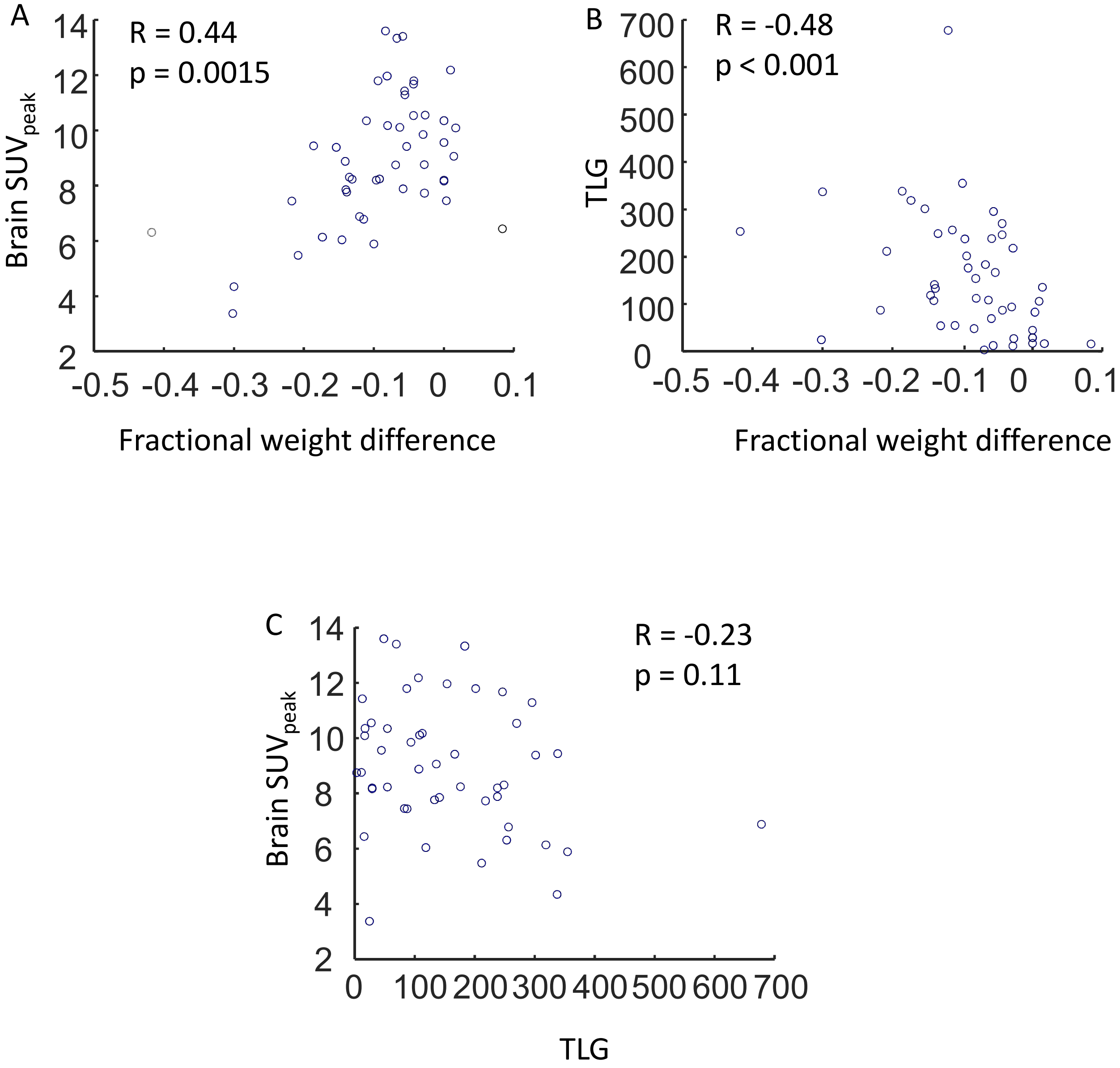

Supplementary Figure S1. Correlation between brain SUV_{peak} and fractional weight difference (A). Correlation between Total Lesion Glycolysis (TLG) and fractional weight difference (B). Correlations beween brain SUV_{peak} and TLG (C). Spearman correlation coefficient and p-value of observing the null hypothesis.
